# Functionalized magnetic nanoparticles: Synthesis, characterization, catalytic application and assessment of toxicity

**DOI:** 10.1038/s41598-018-24721-4

**Published:** 2018-04-19

**Authors:** Mariana Neamtu, Claudia Nadejde, Vasile-Dan Hodoroaba, Rudolf J. Schneider, Liliana Verestiuc, Ulrich Panne

**Affiliations:** 1 Alexandru Ioan Cuza University of Iasi, Interdisciplinary Research Department – Field Science, Lascar Catargi Str. 54, 700107 Iasi, Romania; 2 Bundesanstalt für Materialforschung und -prüfung (BAM), Unter den Eichen 87, 12205 Berlin, Germany; 3 ‘Grigore T. Popa’ University of Medicine and Pharmacy, Department of Biomedical Sciences, Faculty of Medical Bioengineering, M. Kogalniceanu Str. 9-13, 700454 Iasi, Romania; 4 Humboldt-Universität zu Berlin, Department of Chemistry, Brook-Taylor-Str. 2, 12489 Berlin, Germany

## Abstract

Cost-effective water cleaning approaches using improved treatment technologies, for instance based on catalytic processes with high activity catalysts, are urgently needed. The aim of our study was to synthesize efficient Fenton-like photo-catalysts for rapid degradation of persistent organic micropollutants in aqueous medium. Iron-based nanomaterials were chemically synthesized through simple procedures by immobilization of either iron(II) oxalate (FeO) or iron(III) citrate (FeC) on magnetite (M) nanoparticles stabilized with polyethylene glycol (PEG). Various investigation techniques were performed in order to characterize the freshly prepared catalysts. By applying advanced oxidation processes, the effect of catalyst dosage, hydrogen peroxide concentration and UV-A light exposure were examined for Bisphenol A (BPA) conversion, at laboratory scale, in mild conditions. The obtained results revealed that BPA degradation was rapidly enhanced in the presence of low-concentration H_2_O_2_, as well as under UV-A light, and is highly dependent on the surface characteristics of the catalyst. Complete photo-degradation of BPA was achieved over the M/PEG/FeO catalyst in less than 15 minutes. Based on the catalytic performance, a hierarchy of the tested catalysts was established: M/PEG/FeO > M/PEG/FeC > M/PEG. The results of cytotoxicity assay using *MCF-7* cells indicated that the aqueous samples after treatment are less cytotoxic.

## Introduction

Wastewaters are highly complex media containing a wide variety of contaminants. Many of these cannot be easily removed through conventional technological water treatment processes, thus requiring multi-step procedures or expensive techniques^1–4^. One broad class of such hazardous compounds are endocrine disruptive chemicals (EDCs), which include natural hormones and synthetic compounds – personal-care and pharmaceutical products, industrial chemicals, pesticides, etc.^2–8^. Nowadays, the release and accumulation of these compounds into the environment is a subject of great concern since they have the ability to induce long-term damaging effects in living organisms at the level of their endocrine system, by mimicking and interfering with hormone activity^4–7,9,10^. Bisphenol A (BPA) acts as an endocrine disruptor even at low but environmentally relevant concentrations. Since it is a stable hazardous compound, BPA is frequently chosen as a model estrogenic micropollutant from the class of emerging contaminants^8,11–17^.

From the more recently developed water treatment strategies, advanced oxidation processes (AOPs) proved to be powerful for wastewater remediation^1,2,4–12,14–20^. They are quite effective in increasing the biodegradability as well as the detoxification of effluents containing emerging pollutants such as EDCs. The mechanism that makes AOPs effective involves *in-situ* generation of highly reactive chemical species (*E°* = 1.8–2.8 V)^21^ such as hydroxyl (∙OH) radicals, which trigger the non-specific mineralization of target pollutants with high reaction rates and minimal generation of secondary waste, even in mild conditions^15,17,18,22^. A downside of such remediation techniques is that they can often be rather expensive. In order to address this issue, the increasing use of nanomaterials in pollutant control proved to be an effective solution. The rapid progress and benefits of nanotechnology were shown to play a major role in improving water clean-up^23–27^. Recently, magnetic nanocomposites have received great attention and have been extensively used for environmental applications due to their numerous advantages (tunable properties, ease of functionalization, large surface-to-volume ratio, eco-friendly nature, efficient separation due to their magnetic properties, production at large-scale, low cost etc.)^24–26,28–36^. Moreover, such catalysts can be further reused in subsequent treatment cycles^33,34^. From a sustainability point of view, Coleman *et al*.^37^ have investigated the effects of noble metals on the photo-catalytic degradation of several EDCs in water, and have concluded that it is not viable to add expensive metals to photo-catalytic systems in order to enhance the removal of EDCs found in low concentrations in real wastewaters. In this respect, the application of heterogeneous catalysts consisting in surface engineered magnetic nanoparticles (MNPs) represents an inexpensive and efficient approach in water treatment through AOPs^32–34^.

Magnetite (Fe_3_O_4_) is commonly used in the composition of Fenton-like heterogeneous catalysts^17,22,26–30,32–34,38–41^. One strategy for the preparation of magnetically recoverable catalysts is the immobilization of metal ions onto MNPs by modifying their surface with organic molecules (ligands) such as eco-friendly polymers^27,42^. The coating of the magnetic cores is crucial for protection against oxidation and particle aggregation. In the present case, the iron oxide cores were stabilized using low molecular weight polyethylene glycol (PEG) (average *M*_*n* PEG_ = 3,350, Polydispersity Index 1.05–1.10). This provides good nanoparticle stabilization while still maintaining high magnetic properties similar to those of uncoated Fe_3_O_4_. PEG is a synthetic polymer composed of repeating ethylene oxide monomeric subunits and hydroxyl groups at each end of the chain. It is used extensively in biological and medical applications due to its high biocompatibility and hydrophilicity^42–44^. The terminal primary hydroxyl groups are stable at high temperature in the presence of iron, allowing stability and the possibility to further anchor catalytic active species onto the nanosystem’s surface^42,45^. Thus, by using AOPs of photo-Fenton type as pre- or post-treatment sequences in wastewater remediation, the degradation efficiency of micropollutants can be greatly improved; the oxidizing species generated *in-situ* rapidly break down a wide range of harmful organic contaminants into less toxic and less stable substances. Such a procedure is gaining popularity and can replace the multi-step traditional processes while reducing treatment time^46^.

In our study, in order to test BPA degradation efficiency in solution over heterogeneous magnetic catalysts, the surface of pre-synthesized PEG-stabilized Fe_3_O_4_ (M) was modified with either iron(II) oxalate (FeO) or iron(III) citrate (FeC) as source of iron ions for the Fenton-like reaction. The catalytic activity of the final products – three types of magnetic nanocatalysts: M/PEG, M/PEG/FeO and M/PEG/FeC – was evaluated for BPA degradation at laboratory scale in mild conditions. The synthesis procedure, catalyst characterization, pollutant degradation kinetics and cytotoxicity assessment are further discussed. The catalytic behaviours of the tested catalysts were compared, systematically investigating the effect of various reaction parameters. The overall aim was to select the optimum catalyst and operational parameters in reaction for future tests and implementation at pilot-scale.

## Results and Discussion

### Catalyst characterization

**Figure 1:**
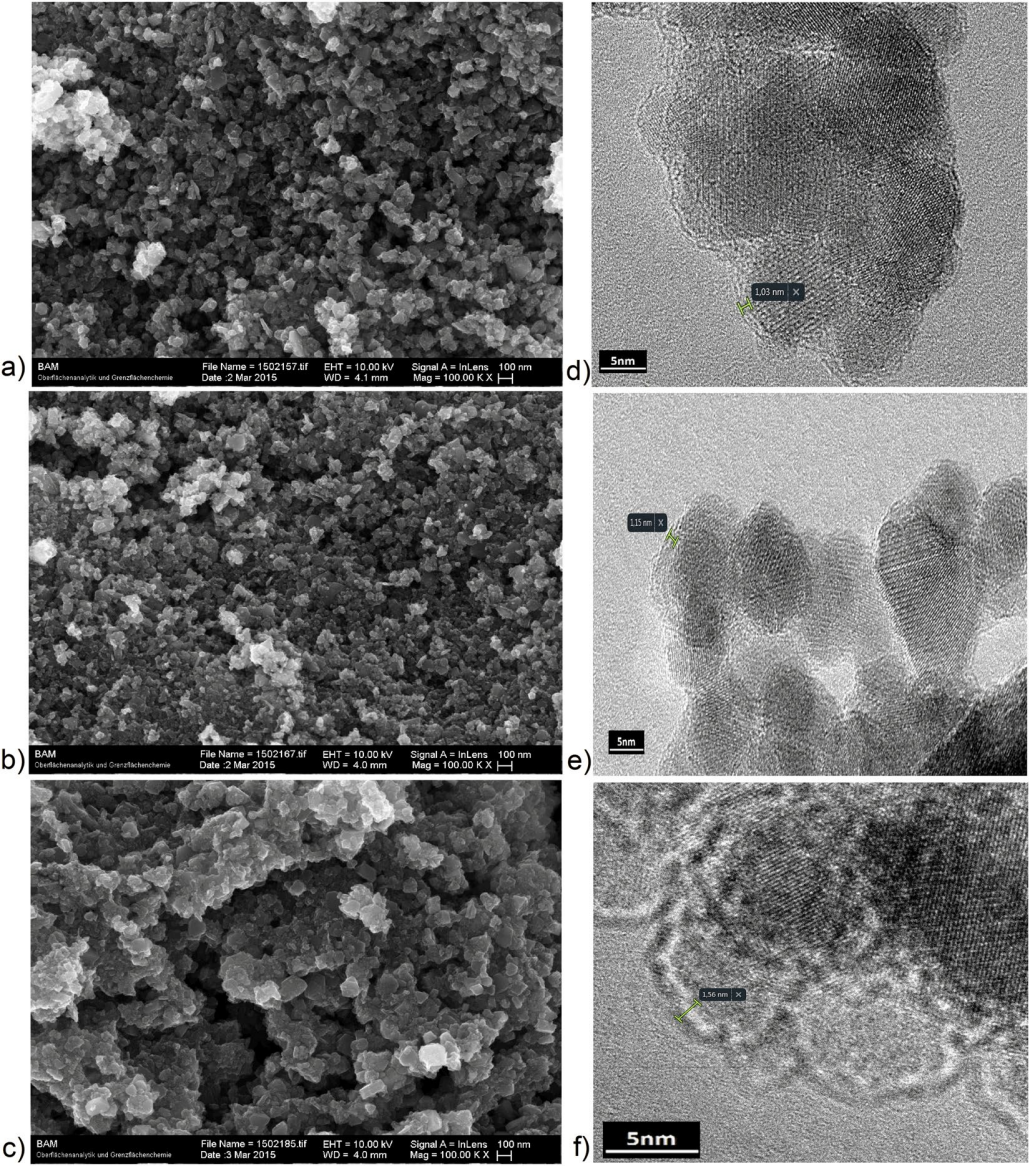
SEM micrographs of (**a**) M/PEG, (**b**) M/PEG/FeO and (**c**) M/PEG/FeC catalysts; HR-TEM micrographs of (**d**) M/PEG, (**e**) M/PEG/FeO and (**f**) M/PEG/FeC catalysts indicating the thin shells of about 1–2 nm thickness.

High-resolution electron microscopy analyses were employed in order to reveal the morphological properties of the catalysts. SEM micrographs (Fig. 1a–c) revealed that the samples consisted of nearly spherical nanosized particles.

HR-TEM images (Fig. 1d–f) confirmed the detailed morphology and nanoscale dimension of the magnetic composites. The polydispersity and spherical-like shape of individual particles, with sizes up to 25 nm, are typical features for MNPs synthesized by co-precipitation^44,47–50^. The ultrathin coating up to 2 nm surrounding the magnetic cores was also evidenced by other authors^44,50,51^.

The elemental composition of the three types of catalysts was analysed by EDX at different beam voltages, which led to the identification of the main elements in the catalyst structure: C, O and Fe (Fig. S1 in Supplementary Information (SI) file).

The FT-IR spectral analysis was also used for catalyst characterization in order to retrieve information related to the surface chemistry. FT-IR spectra of the magnetic catalysts were analysed comparatively to those of the individual component materials, as shown in Fig. S2 in SI file.

**Figure 2:**
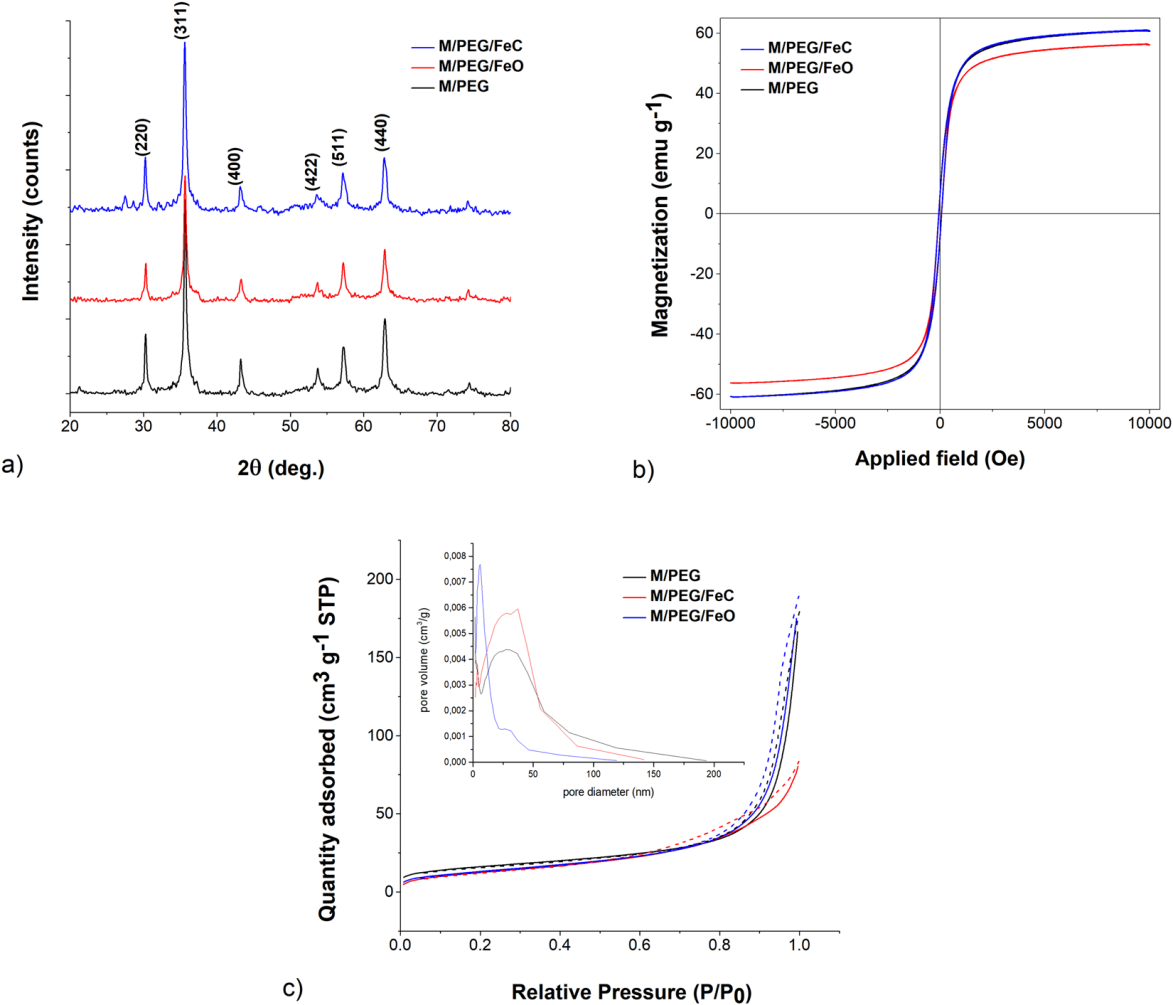
(**a**) The XRD patterns of synthesized magnetic catalysts; (**b**) Magnetization curves of the studied catalysts; (**c**) The nitrogen adsorption-desorption isotherms of the three catalysts. Inset: the pore size distribution curves corresponding to each catalyst.

The structure phase and average core size of the synthesized catalysts were analysed based on the recorded XRD patterns of the obtained powdered samples. As depicted in the diffractograms in Fig. 2a, magnetite was the dominant crystalline phase in all samples exhibiting the corresponding typical spinel cubic structure of iron oxide (confirmed by the reflection planes assigned to the diffraction peaks). The narrow and high intensity diffraction peaks of the obtained XRD patterns indicate that all manufactured materials consist of grains with high crystallinity and increased crystallite sizes (see Table S1 in SI file).

The studied magnetic catalysts showed high saturation magnetization values of 60.74 emu g^−1^ for M/PEG, in agreement with scientific reported data^47,48^, of 56.20 emu g^−1^ for M/PEG/FeO and 60.70 emu g^−1^ in the case of M/PEG/FeC (Fig. 2b). The iron(II) oxalate functionalized PEGylated magnetite seems to exhibit minor decrease in the saturation magnetization compared to the other two catalysts. The good magnetic properties show that the prepared catalysts can be easily retrieved from solution using an external magnet without loss of the magnetic material.

The N_2_ adsorption-desorption isotherms recorded for all three materials (Fig. 2c) can be classified as type IV according to IUPAC. This indicates the predominance of mesoporous structure in the studied catalysts^17,49^. The first two materials (M/PEG and M/PEG/FeO) present narrow hysteresis loops at a relative pressure in the 0.70 to 0.99 range, while in the case of M/PEG/FeC catalyst, the pressure range extends from 0.50 to 0.99. The inset in Fig. 2c provides further details regarding the pore size distribution curves resulting from the corresponding nitrogen isotherms measurements.

As it can be observed, the isotherms, as well as the pore size distribution curves, of the M/PEG and M/PEG/FeO materials are rather different from those of M/PEG/FeC: the former mentioned catalysts possess larger pore diameters and volumes, together with higher BET specific surface areas, as compared to the latter (Table 1). By comparing the FeO and FeC catalysts between them, one can observe similar BET surface area values, which is in agreement with the reported data^17,28,49^.

**Table 1:** Results of BET method applied for the synthesized catalysts.

Catalyst type	BET surface area (m^2^ g^−1^)	BJH pore size (nm)	Pore volume (cm^3^ g^−1^)
M/PEG	56.39	19.6	0.28
M/PEG/FeO	48.13	24.4	0.29
M/PEG/FeC	46.58	11.1	0.13

### Evaluation of catalytic activity of the studied catalysts

**Figure 3:**
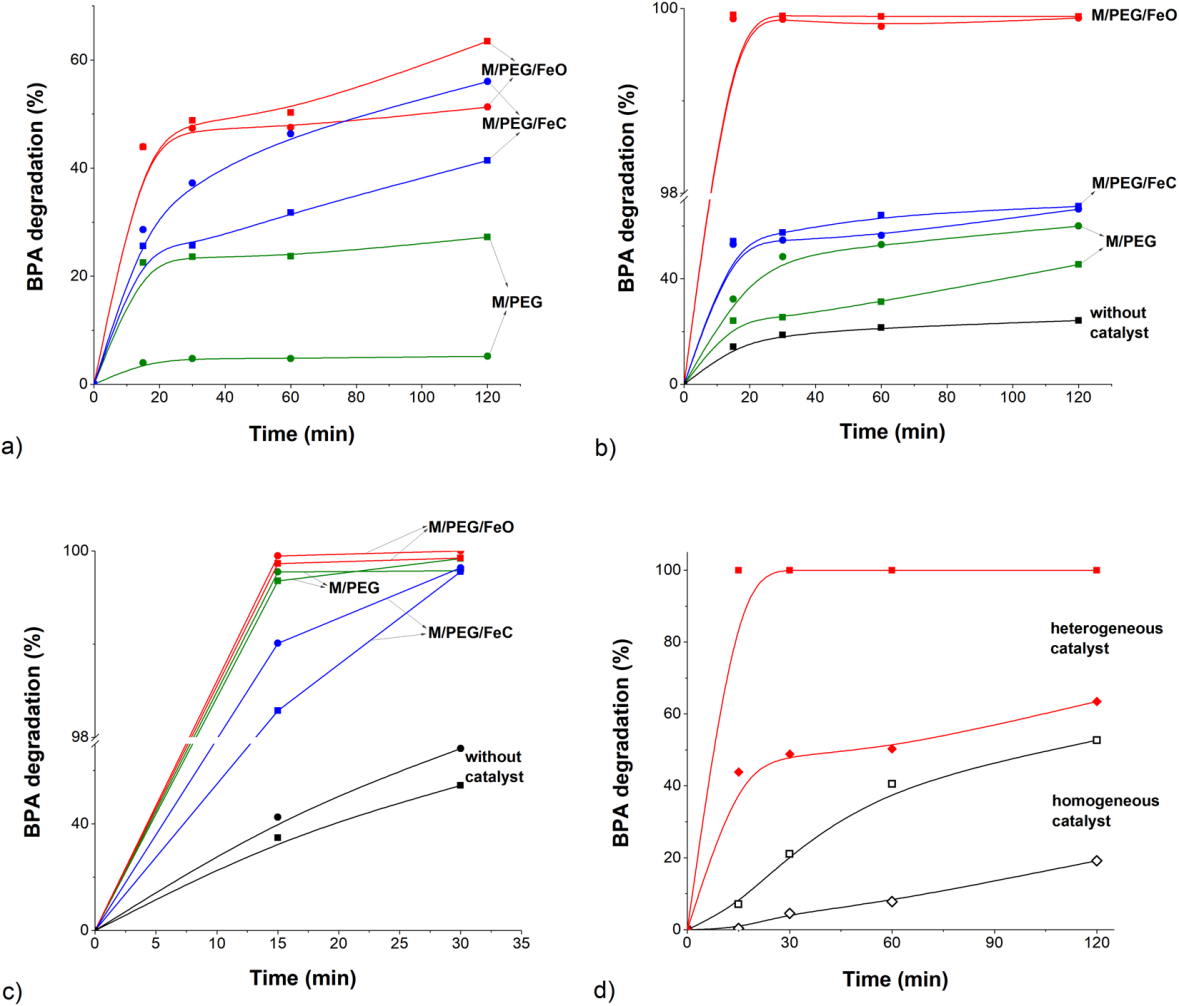
Degradation of BPA in aqueous solution: the effect of (**a**) catalyst concentration (◼ −1.0 g L^−1^; ● −1.5 g L^−1^); (**b**) hydrogen peroxide dosage (◼ −10 mmol L^−1^; ● −20 mmol L^−1^); (**c**) UV-A exposure on the BPA conversion over each catalyst (◼ −UV-A; ● −UV-A/10 mmol L^−1^ H_2_O_2_); (**d**) Comparison between the efficiency of the homogeneous (empty symbols) and heterogeneous (filled symbols) oxidation of BPA (♦, ◊ – in the absence of H_2_O_2_; ◼, □ – in the presence of 10 mmol L^−1^ H_2_O_2_; black – 8.36 × 10^−3^ g L^−1^ FeO; red −1.0 g L^−1^ of M/PEG/FeO). Initial conditions: 0.5 μmol L^−1^ of BPA, pH 6.6, *t* = 25 °C, 1.0 g L^−1^ of catalyst (**b**,**c**).

The catalytic behaviour of the prepared magnetic catalysts was assessed by evaluating the efficiency of BPA degradation in aqueous solution. In order to make the process sustainable, the most appropriate operational parameters must be established with minimum use of chemicals while ensuring the highest efficiency for pollutant degradation. The key features of Fenton-like oxidation processes lie in the reaction characteristics (dosage of organic and inorganic constituents, pH, temperature) and their optimal combination. Here, BPA oxidation was evaluated using the three as-synthesized heterogeneous catalysts in mild conditions (initial pH 6.6, room temperature). The rate of BPA degradation in solution during 120 minutes of reaction was monitored for the following operational conditions: catalyst dosage, hydrogen peroxide (H_2_O_2_), UV-A exposure (Fig. 3). All reactions were found to follow the first-order kinetics law.

#### Effect of catalyst dosage

Two catalyst concentrations were first tested aiming to establish the optimum dosage for which the fastest BPA degradation rate is obtained. Figure 3a shows that a concentration of 1.0 g L^−1^ of catalyst is able to trigger the degradation of BPA in solution. The M/PEG/FeO catalyst has the highest reaction rate constant (0.207 min^−1^) among all the tested materials in the above conditions; specifically, M/PEG/FeO was able to decompose about 50% of BPA in 30 minutes and over 60% in 120 min. This could probably be explained by the behaviour of different iron species. The other two nanomaterials exhibit significantly lower and alike values of the rate constant in the same reaction conditions (Table 2).

**Table 2:** Pseudo-first-order reaction constants (min^−1^).

Process	Without catalyst	Catalyst type		
		M/PEG	M/PEG/FeO	M/PEG/FeC
1.0 g L^−1^	—	0.0170	0.2071	0.0174
1.5 g L^−1^	—	0.0022	0.1747	0.0142
10 mmol L^−1^ H_2_O_2_	0.0040	0.0184	0.4846	0.0520
20 mmol L^−1^ H_2_O_2_	—	0.0260	0.4525	0.0505
UV-A	0.0266	0.3824	0.4399	0.2711
UV-A/10 mmol L^−1^ H_2_O_2_	0.0308	0.4062	0.5016	0.3076

Subsequent increase of catalyst concentration to 1.5 g L^−1^ leads to a lower degradation efficiency of BPA, with a lower rate constant of 0.175 min^−1^ for M/PEG/FeO as compared with the previous case, probably due to the agglomeration tendency of the MNPs at concentrations higher than 1.0 g L^−1^. This may lead to the blocking of the available active sites on the material surface, reducing the specific surface area and slowing down the pollutant degradation^49^. Therefore, the 1.0 g L^−1^ catalyst concentration was maintained in the following experiments. These results suggest that a molecular oxygen activation process may occur in solution generating Reactive Oxygen Species (ROSs) in the presence of iron and further triggering the BPA oxidation and break-down over the catalysts in the absence of hydrogen peroxide and/or UV irradiation^38,52,53^. Similar results were reported by Xu and Wang (2012)^49^ when using the same concentrations of uncoated MNPs for the degradation of 2,4-dichlorophenol.

#### Effect of hydrogen peroxide concentration

The selection of an optimal H_2_O_2_ concentration for BPA degradation is important not only since H_2_O_2_ plays the role of oxidizing agent in the Fenton-like process, but also considering the procedure’s cost. As evidenced by the data plotted in Fig. 3b, when adding 10 mmol L^−1^ of H_2_O_2_, the oxidation process leads to fast BPA degradation in the first 15 minutes, followed by a slower degradation, probably as a result of the hydroxyl radical consumption^11,17^. Specifically, the catalytic oxidation of BPA was completed in about 15 minutes over M/PEG/FeO with the highest rate constant of 0.485 min^−1^, in the given reaction conditions. When using the M/PEG/FeC material, only about 54% of BPA conversion was achieved in the same time interval (15 min), with a significantly lower reaction rate constant of 0.052 min^−1^. By further increasing the H_2_O_2_ concentration from 10 mmol L^−1^ to 20 mmol L^−1^, no significant improvement of oxidation efficacy was observed (Fig. 3b, Table 2). Other studies reported efficient BPA mineralization following pseudo-first-order reaction using magnetite-based catalysts together with various types of AOPs methods. Huang *et al*.^17^ obtained only a small percent of BPA degradation when using 0.585 g L^−1^ uncoated MNPs and 160 mmol L^−1^ H_2_O_2_ at pH 7, in an 8 h interval; the removal efficiency significantly increased to 98.1% when adding ultrasound exposure to the reaction medium; by increasing the H_2_O_2_ concentration, the degradation efficiency was diminished due to the competition of excess H_2_O_2_ at the catalyst surface, limiting the decomposition of the pollutant, similarly to our results in the current study.

The kinetic tests carried out in the presence of H_2_O_2_ alone showed the low efficiency of BPA oxidation (Fig. 3b, Table 2): only 24% conversion of BPA was obtained with 10 mmol L^−1^ of H_2_O_2_ in aqueous solution, within the tested time of 120 min. Therefore, the following experiments using UV-A irradiation of the BPA aqueous samples containing 1.0 g L^−1^ of catalysts were performed in the presence of 10 mmol L^−1^ of H_2_O_2_.

#### Effect of UV-A irradiation

The photo-oxidation experiments evidenced fast total BPA degradation in 15 min over the M/PEG/FeO catalyst, and nearly complete conversion in 30 minutes over the other two materials (Fig. 3c, Table 2). The UV-A radiation in the presence of H_2_O_2_ accelerated the BPA degradation process from 0.485 min^−1^ to 0.502 min^−1^ when using the M/PEG/FeO catalyst. Moreover, by applying UV-A irradiation, a significant increase in the BPA reaction rate constants occurred in the case of M/PEG/FeC (from 0.052 min^−1^ to 0.308 min^−1^) and M/PEG (from 0.018 min^−1^ to 0.406 min^−1^) as compared with the previous reaction conditions (without UV-A irradiation). As depicted in Fig. 3c, one can observe that UV-A exposure alone leads to enhanced BPA removal over all tested catalysts in 30 min of reaction, although not as fast as the simultaneous application of H_2_O_2_ and UV-A (Table 2).

The experiments performed with iron(II) oxalate as homogeneous catalyst show the superior efficiency of heterogeneous process as opposed to the homogeneous one (Fig. 3d), as well as the crucial role of the properties of MNPs. (The experiments in homogeneous phase were carried out in the same experimental conditions as the heterogeneous one, in the absence of UV-A exposure).

By using ferromagnetic catalysts in organic pollutant degradation, either in the absence or presence of H_2_O_2_ and UV-A, a certain degree of BPA mineralization takes place in aqueous solution; this is possible due to ROSs – i.e. singlet oxygen (^1^O_2_), superoxide radical (∙O^2−^), hydroxyl radicals (∙OH) - produced via single-electron reduction pathway which activates the molecular oxygen, leading to the oxidation of persistent contaminants^24,54^. Efficient BPA conversion was also reported by Rodriguez *et al*.^55^ after 120 min of reaction: 92.3% over Fe_3_O_4_/oxalic acid/H_2_O_2_/UV-A and 100% over Fe_3_O_4_/oxalic acid/TiO_2_/H_2_O_2_/UV-A systems and with lower efficiencies in the absence of oxalic acid ([BPA]_0_ = 10^−5^ mol L^−1^; [Fe_3_O_4_] = 0.15 g L^−1^; [H_2_O_2_]_0_ = 5 × 10^−4^ mol L^−1^; [TiO_2_] = 0.1 g L^−1^; [Oxalic acid]_0_ = 2 × 10^−4^ mol L^−1^, pH 3). Li *et al*.^56^ have also revealed that the Fe-oxalate species on the surface of maghemite are responsible for enhanced degradation of BPA during classical photo-oxidation treatment (iron ions, UV light, acidic pH)^56^. However, our study offers an improved approach for BPA removal by using the iron(II) oxalate immobilized on the MNPs in mild conditions (near-neutral pH) during the photo-oxidation procedure.

In the current investigation, as it can be concluded from Table 2, the best conditions for BPA degradation over the studied catalysts were 1.0 g L^−1^ of catalyst, 10 mmol L^−1^ H_2_O_2_ with UV-A irradiation. The highest rate constants were obtained when using the M/PEG/FeO catalyst. It is known that Fe^2+^ species (FeO) are more active in Fenton reaction and hence, a high activity is expected. However, regarding M/PEG/FeC contribution to BPA oxidation, first Fe^3+^ is converted into Fe^2+^ by the protons of the reaction medium, then Fe^2+^ reacts with H_2_O_2_ generating ∙OH radicals (Fenton-like process). The obtained results in this study show that, although the specific surface area can play a role in catalyst reactivity, the iron ions immobilized on catalyst surface are significantly responsible for their catalytic performance in BPA degradation (since the specific surface areas of M/PEG/FeO and M/PEG/FeC have similar values). Although reports on oxalate capped iron nanomaterials are limited, their numerous advantages (eco-friendly nature, excellent reusability, low-cost, etc.) and high efficiency in the degradation of a wide variety of refractory pollutants were revealed only recently^28,38,39,57^.

#### Cytotoxicity tests

We have shown that following the BPA degradation through AOPs of Fenton type, a rapid pollutant conversion is achieved; however, during such water treatments, a great concern is related to the subsequent potential toxicity of the degradation products in the reaction medium, since BPA is converted into less stable intermediate species. Since many other studies report on BPA oxidation intermediates, no elucidation of transformation products has been carried out in the present study. It is known that the mechanism of BPA mineralization using AOPs of (photo)Fenton type involves the generation of ∙OH radicals, responsible for breaking-down the target micropollutant. The two benzene rings in BPA are cleaved during the initial oxidation reaction, since the electron density of each aromatic ring is increased by the electron-donating hydroxyl groups. In the initial oxidation reaction, following the attack by ∙OH and cleavage of phenyl groups, BPA is converted into a series of intermediate products including 4-isopropylphenol, 1,4-benzoquinone, p-hydroquinone, 4-(1-hydroxy^−1^-methyl-ethyl)-phenol, phenol. These compounds are further transformed into short-chain aliphatic compounds, and finally, into CO_2_ due to a complex oxidative ring-opening reaction at the level of C‒C bonds^11–14,58^. In our study, some intermediates (1,4-hydroquinone and phenol) were detected in low concentration during BPA conversion. More details are provided in SI file, Fig. S3. This is in agreement with other reports on BPA degradation using AOPs of Fenton type^11–14,58^. Regarding the intermediates toxicity, Makarova *et al*.^13^ assessed the toxicity of the BPA degradation products for zebrafish embryos and found that hydroquinone (20 mg L^−1^) had no toxic effects in the development of zebrafish embryo. Olmez-Hanci *et al*.^14^ found that, when using raw freshwater samples, no toxicity effects of the resulted intermediate products were detected on *Vibrio fischeri* organisms after BPA degradation, as opposed to the oxidation experiments performed on model BPA aqueous solutions; they have concluded that this was probably due to additional organic and inorganic species present in natural water matrix, which seem to significantly influence the process and affect the BPA mineralization by AOPs.

**Figure 4:**
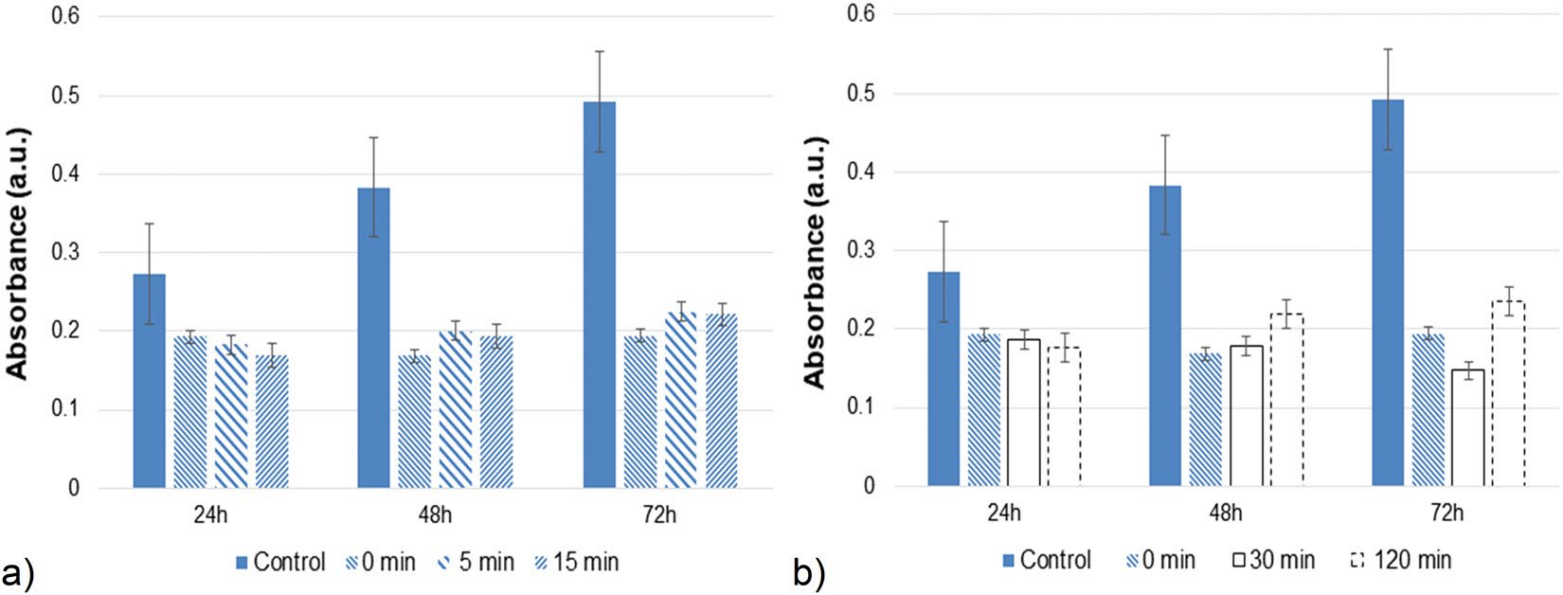
Cytotoxic assessment of samples after treatments: in the presence of 10 mmol L^−1^ H_2_O_2_ (**a**) and in the absence of H_2_O_2_ (**b**). Initial conditions: 0.5 μmol L^−1^ of BPA, 1.0 g L^−1^ of M/PEG/FeO catalyst, pH 6.6, *t* = 25 °C, sample dilution of ¼.

Degradation of BPA and cessation of its estrogenic activity are the goals in water treatment. A *MCF-7* cell viability assay was used to determine if the samples exhibit cytotoxic effects after the proposed BPA removal treatment. The cell viability data (Fig. 4) showed that, at 72 h of incubation, when the BPA was completely removed after 15 minutes of treatment over M/PEG/FeO catalyst in the presence of 10 mmol L^−1^ H_2_O_2_ (Fig. 4a), the cytotoxicity of metabolically active cells slightly increased, indicating that after treatment the samples are less cytotoxic. The samples at 120 min of treatment in the absence of hydrogen peroxide elicited similar behaviour (Fig. 4b). The still low absorbance compared to control could be related to the identified intermediates that have been detected to exhibit cytotoxic effect (SI file, Fig. S4).

These results evidenced that the *MCF-7* cell viability assay is highly sensitive, reproducible, rapid, has no variable background issue, all of which facilitate its application in screening the cytotoxicity of various aqueous samples.

#### Reusability and stability tests

Catalysts should also be stable under various reaction conditions and should maintain their activity upon repeated use. The investigated catalysts can be easily separated from aqueous solution, thus allowing their reuse in consecutive water treatment cycles. Thus, kinetic experiments of catalyst reusability were performed under the above-established optimum operational conditions (1.0 g L^−1^ of catalyst, 10 mmol L^−1^ H_2_O_2_, in the absence of UV-A exposure) using M/PEG/FeO, due to its highest catalytic activity. After two consecutive runs, the catalyst still exhibits a good activity, despite a slight decrease in pollutant degradation from 100% to 87.25%, after 120 min under above experimental conditions, whereas after three consecutive runs the BPA degradation decreases from 100% to 78%.

The stability of the iron-based catalysts in terms of non-leaching of iron remains a challenge. In the case of the catalyst with the highest activity (M/PEG/FeO), the iron concentration level in the supernatants was examined at different time intervals during 120 min of reaction (Fig. S5 in SI file). During BPA oxidation, the total iron concentration in solution tends to increase with reaction time reaching 2.45 mg L^−1^ after 120 min (well below the EU directives of <5.0 mg dm^−3^ for surface waters)^59^. This indicates that iron ions from FeO get from the catalyst surface into the reaction medium during the pollutant removal process, and thus promote the occurrence of homogeneous Fenton reaction in the bulk solution. Similar results were obtained by Xu and Wang^49^ investigating the catalytic performance of MNPs in the removal of 2,4-dichlorophenol from aqueous solutions. Other studies have pointed out that the enhanced leaching of iron in the reaction medium during Fenton oxidation can be attributed to the occurrence of reducing agents in solution^55,60^. Such reductants are, among others, hydroquinone and oxalic acid (BPA by-products). It has been shown^55,60^ that reductants can destabilize the iron at the catalyst surface and induce its detachment as Fe^2+^ which then diffuses in bulk solution. Future studies are required to overcome iron leaching from the catalyst surface, while extending the catalyst ability to maintain high performance during several water treatment cycles. The obtained data reveal that the sensitized materials could be promising catalysts also for other pollutants removal once the optimum conditions in batch reactors and at pilot-scale are established. Constant improvement of the existing wastewater treatment methods needs to be sought to ensure better quality of waters and to protect the environment.

## Conclusions

The current study presents an alternative eco-friendly method for efficient removal of the recalcitrant endocrine disruptor and model compound Bisphenol A (BPA), from wastewaters. The kinetic tests performed using sensitized iron-based magnetic catalysts revealed the high efficiency of BPA degradation. The highest BPA degradation rates were observed for PEGylated magnetite modified with iron(II) oxalate, which revealed an excellent catalytic performance even in the absence of UV irradiation. Complete fast BPA removal was achieved over such catalyst (1.0 g L^−1^) in less than 15 minutes of reaction time, in the presence of 10 mmol L^−1^ H_2_O_2_ and UV-A irradiation. The above findings suggest the high suitability of iron oxalate-based magnetic catalysts for the efficient BPA degradation. They could be promising candidates for the removal of a wide range of persistent micropollutants from waters. We consider that such catalysts could be successfully applied to precede or follow the main wastewater treatment sequence, in mild conditions, simplifying the procedure and reducing processing time and costs.

## Methods

### Reagents and chemicals

Bisphenol A, PEG, iron(II) oxalate dihydrate, iron(III) citrate, and all other chemicals (Sigma-Aldrich) were used as received, being high purity reagents. Ultrapure water (0.055 µS cm^−1^) was used in all experimental procedures and was produced using an Evoqua LaboStar Pro TWF UV system.

### Preparation of the catalysts

For magnetite (M) nanoparticle synthesis, the conventional wet chemical route was chosen in this study due to its low cost and efficiency. In short, the co-precipitation method used in here according to Massart’s method^61,62^, consisted in mixing aqueous solutions of iron(III) and iron(II) salts (in a 2:1 stoichiometric ratio) together with a highly alkaline solution (1.75 mol L^−1^ NaOH). More detailed information on the preparation protocol can be obtained from our previous studies^38,39^. Polymer encapsulation of the obtained MNPs was carried out immediately after Fe_3_O_4_ synthesis. The stabilization of the as-obtained magnetite (~4 g) with 1% w/v low-molecular weight PEG was performed at constant temperature (60 °C) under continuous rapid mechanical stirring for 1 h. Following subsequent purification of the PEGylated magnetite (by alternated washing the product with ultrapure water and absolute ethanol with magnetic separation), part of the product (M/PEG catalyst) was vacuum-dried at 50 °C. The rest of the fresh wet material was kept for further functionalization with the photo-active compounds at the same molarity, i.e. 39.5 mmol L^−1^: either FeO or FeC. In this procedure, the M/PEG/FeO catalyst was prepared by gradually adding 0.5 g FeO to 1.75 g of M/PEG under continuous mechanical stirring at 55 °C for 2 h. The same protocol was followed to obtain the M/PEG/FeC catalyst, for which 0.73 g FeC was mixed with 1.75 g of M/PEG, in the same above-mentioned experimental conditions. The obtained catalysts were finally purified, then vacuum-dried at 50 °C.

### Catalyst characterization

All three types of catalysts were investigated by conventional characterization techniques in order to establish their physical-chemical properties. The catalyst morphology and elemental composition were examined with a high-resolution scanning electron microscope (SEM, Supra 40 Carl Zeiss, Oberkochen, Germany), having a Schottky field emitter, and a silicon drift detector (SDD) energy dispersive X-ray spectrometer (EDS) (Bruker XFlash® 5010, Berlin, Germany). High resolution transmission electron microscopy (HR-TEM) (Zeiss Libra 200 MC TEM/STEM electron microscope, possessing a SDD EDS spectrometer, model Bruker XFlash® 5030, Berlin, Germany), was also employed in order to get a more detailed view of the samples’ size and inner structure.

Fourier transform infrared (FT-IR) spectroscopy (Jasco 660 Plus spectrometer) was applied on the powder samples prepared in KBr discs to evaluate the surface modification of MNPs, in the 400–4000 cm^−1^ range at room temperature (resolution of 4 cm^−1^).

The crystalline phase of the synthesized catalysts was evaluated by X-ray diffraction (XRD) in the range of 2*θ* from 20° to 80°, using a Bruker AXS D8-Advance diffractometer (Cu-Kα radiation, *λ* = 0.1541 nm).

The magnetization curves of the prepared catalysts were obtained on a vibrating sample magnetometer (VSM) (model MicroMag 2900/3900) at room temperature.

The catalyst surface properties were analysed by N_2_ adsorption-desorption isotherms using the Brunauer–Emmett–Teller (BET) method (at 77 K, relative pressure (*P*/*P*_0_): 0–0.99), on a Micromeritics ASAP 2020^TM^ Physisorption system (Norcross, USA); the Barrett–Joyner–Halenda (BJH) method was used to calculate the sample pores’ mean diameter and volume. All samples were degassed under vacuum at 105 °C for 4 h before the nitrogen adsorption measurements.

### Catalytic BPA oxidation experiments

The kinetic experiments were performed in mild conditions (initial pH 6.6, ambient temperature 25 ± 2 °C) at laboratory scale. The initial concentration of BPA in all experiments was 0.5 µmol L^−1^. The reaction was initiated after reaching equilibrium in solution. Furthermore, the solution was analysed after stabilization occurred, in order to confirm that no adsorption process takes place at catalyst surface. The wet chemical oxidation tests on BPA aqueous solutions were carried out on a SB3 overhead rotator (VWR International, Darmstadt, Germany). To study the effect of catalyst concentration, 1.0 and 1.5 g L^−1^ of catalyst were evaluated in the BPA degradation experiments at various time intervals with total contact time of 120 min. From a 30% H_2_O_2_ standard solution, two concentrations of 10 and 20 mmol L^−1^ were used in order to assess their effects on the catalytic efficiency. The photo-chemical tests using UV-A irradiation of the aqueous samples were performed in a Heidolph Titramax 100 orbital shaker equipped with an UV-A simulator (three parallel Xe lamps, 290 ≤ *λ* ≤ 400 nm, 40 W m^−2^, ATLAS Material Testing Solution, Germany): the diameter of irradiated vessel was 3.5 cm, the irradiated surface area was 9.62 cm^2^, the irradiation path-length was 1.4 cm, the total reaction volume was 8 mL for each sample, and photon flux of the UV simulator system was 2.13 × 10^−7^ Einstein s^−1^. More details are provided in our previous publications^38,63^. During kinetic experiments, aliquots from each sample were withdrawn regularly, immediately filtered (0.22 µm Teflon membrane) and submitted for HPLC analysis (HPLC–MS/MS system) in order to measure the BPA concentration in each tested sample. An 1100 HPLC workstation (Agilent Technologies, Waldbronn, Germany) was used for analyses, coupled to an API 4500 TSQ triple stage quadrupole mass spectrometer (ABSciex, Darmstadt, Germany). The detailed description of the experimental set-up and method of analysis can be found in our previous study^63^.

The iron level in solution was determined using a GF-HR-GS atomic absorption spectroscopy apparatus (AAS) with platform contrA600 (Analytik Jena), optimized and calibrated using stock solutions diluted from a certified standard solution (Merck) in ultrapure water with 0.05% HNO_3_.

The experiments were carried out in triplicate for each catalyst; the calculated relative standard deviation was less than 3%.

### Cytotoxicity assays

A *MCF-7* cell viability assay was used to determine if the BPA aqueous samples, after treatment, exhibit cytotoxic effects. The detailed procedure is described in Balan *et al*.^64^.

### Data Availability

All data generated or analysed during this study are included in this published article (and its Supplementary Information files).

## Electronic supplementary material

Supplementary Information
