# Supplementary material for: Functionalized magnetic nanoparticles: Synthesis, characterization, catalytic application and assessment of toxicity
Source: Sci Rep. 2018 Apr 19;8:6278. doi: 10.1038/s41598-018-24721-4 (PMC5908962; doi:10.1038/s41598-018-24721-4)
Supplement: Supplementary file 1 — Supplementary Information [file 41598_2018_24721_MOESM1_ESM.pdf]

Supplementary Information

Functionalized magnetic nanoparticles: Synthesis, characterization, catalytic application and assessment of toxicity

Mariana Neamtu,[a*] Claudia Nadejde,[a] Vasile-Dan Hodoroaba,[b] Rudolf J. Schneider,[b] Liliana Verestiuc,[c] and Ulrich Panne[b,d]

[a]*Alexandru Ioan Cuza University of Iasi, Interdisciplinary Research Department – Field Science, Lascar Catargi Str. 54, 700107 Iasi, Romania. *E-mail: mariana.neamtu@uaic.ro*

[b]*Bundesanstalt für Materialforschung und -prüfung (BAM), Unter den Eichen 87, 12205 Berlin, Germany*

[c]*'Grigore T. Popa' University of Medicine and Pharmacy, Department of Biomedical Sciences, Faculty of Medical Bioengineering, M. Kogalniceanu Str. 9-13, 700454 Iasi, Romania*

[d]*Humboldt-Universität zu Berlin, Department of Chemistry, Brook-Taylor-Str. 2, 12489 Berlin, Germany*

Catalyst characterization.

EDX results

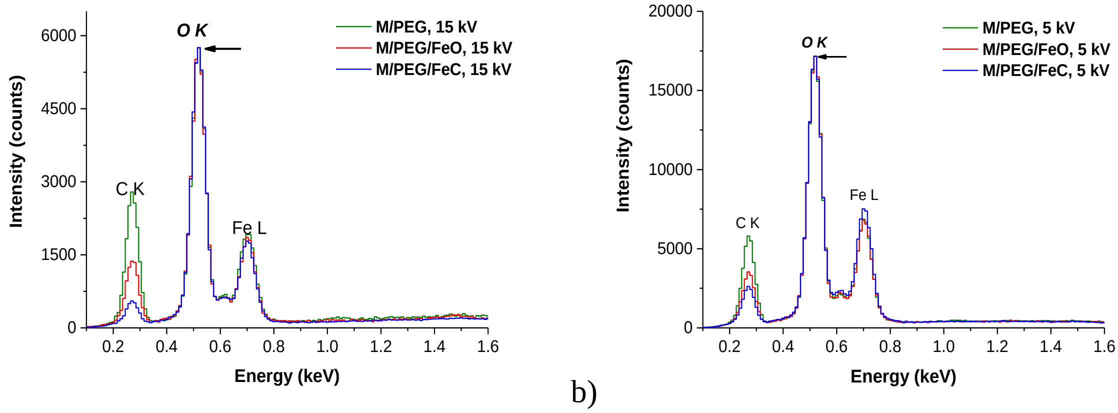

a) b)

Figure S1. EDX spectra at (a) 15 kV and (b) 5 kV of each catalyst, normalized on the intensity of oxygen peak.

The findings at 15 kV (Fig. S1a) were further confirmed by the EDX spectra measured at 5 kV (Fig. S1b). The EDX data confirms the significant fraction of carbon in the M/PEG sample, while the other two catalysts exhibit similar iron to oxygen intensity ratios. The higher carbon content in M/PEG sample could be probably explained by the presence of higher PEG content in external layer of the catalyst in comparison with other two catalysts, which have been further functionalized with an additional layer of catalytically active material (either FeO or FeC).

FT-IR measurements

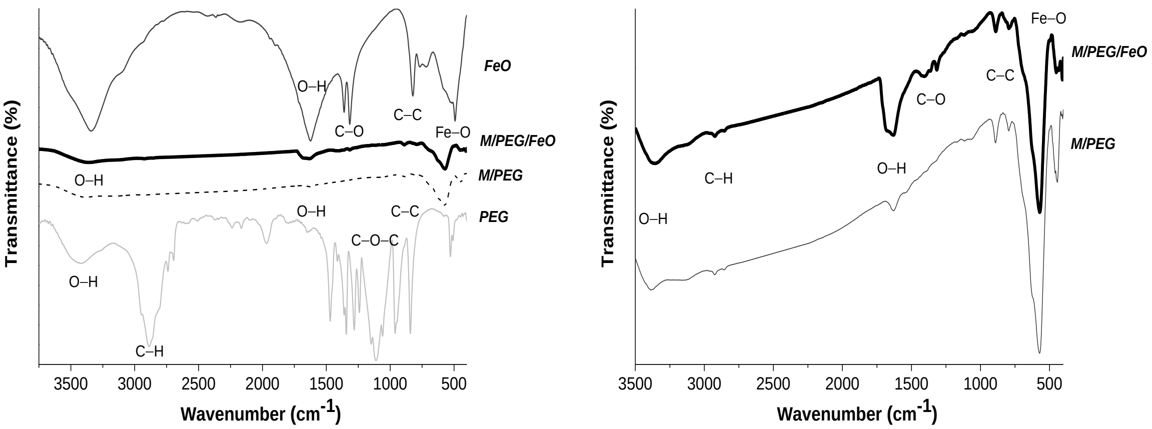

a)

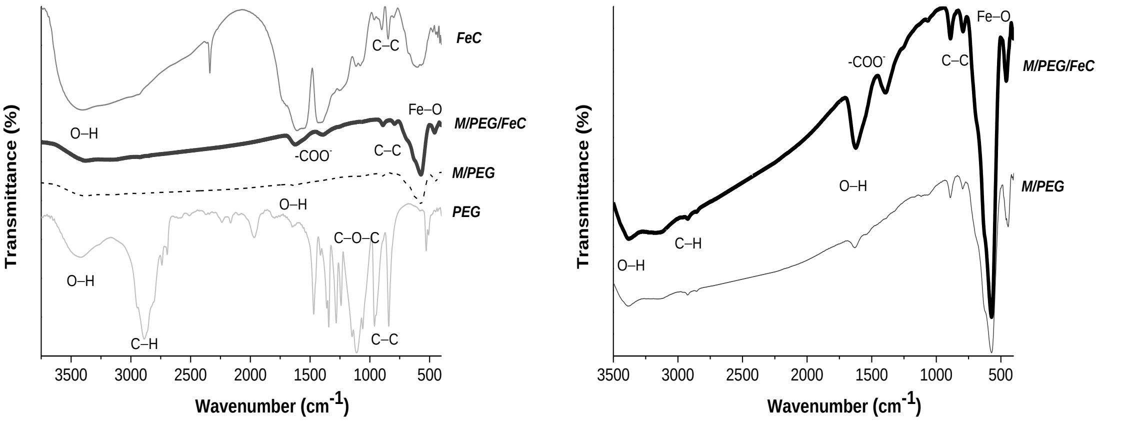

b)

Figure S2. Compact (left) and enlarged (right) FT-IR spectra of the magnetic catalysts – M/PEG/FeO (a) and M/PEG/FeC (b) – and of the individual pure materials.

All catalysts exhibit strong metal-oxygen vibration bands in the lower wavenumber region: one around 570 cm^{-1} resulting from the Fe stretching vibrations at tetrahedral site, while the Fe–O

vibration around 405 cm^{-1} can be assigned to the octahedral site of magnetite[1-3]. Since an ultrathin polymeric shell is surrounding the magnetic cores, the magnetite-PEG interaction is not evidenced by intense well-defined vibration peaks. However, weak C–C vibration modes of PEG in the region 965-840 cm^{-1} were shifted at lower wavenumbers in the M/PEG spectrum, being identified at 795.5 cm^{-1} and 891.9 cm^{-1} [2-4]. The strong peak at 1,110 cm^{-1} in the PEG FT-IR spectrum is assigned to the C–O–C stretch of PEG ether groups; together with the bending of C–H units in PEG backbone in the region 1,470-1,340 cm^{-1}, they are extremely weak in M/PEG, M/PEG/FeO and M/PEG/FeC spectra, indicating dipole-cation interactions between PEG and magnetite[5]. The bending and stretching vibrations of the surface hydroxyl groups, identified at 1,650 cm^{-1} and 3,422 cm^{-1} in PEG spectrum and, respectively, at 1,622.8 cm^{-1} and 3,342 cm^{-1} in FeO, and at 3,416.3 cm^{-1} in FeC, are shifted in M/PEG, M/PEG/FeO and M/PEG/FeC due to the intermolecular hydrogen bonding. In the region from 750 to 1,700 cm^{-1}, it can also be seen that the specific vibrating modes from either FeO or FeC are present on the catalyst surface, confirming the binding of either iron(II) oxalate or iron(III) citrate to the M/PEG nanoparticles.

XRD characterization – determination of crystallite average size corresponding to each magnetic nanocatalyst. The values of grain average diameters (d_{XRD}) in the catalyst composition are given in Table S1; they were estimated from the recorded XRD pattern of each sample using Scherrer equation: $d_{XRD} = K\,\lambda/(\beta \cos\theta)$, where $\lambda = 0.15406$ nm is the incident X-ray wavelength, K is the particle shape factor (0.94 for magnetite), β is given by the full width at half-maximum of the (3 1 1) diffraction reflection and θ is the corresponding diffraction angle (here, $2\theta = 35.6°$). The data are in agreement with the SEM/HR-TEM results.

Table S1. The crystallite average size corresponding to the Fe_3O_4 cores of each catalyst (calculated with Scherrer equation from the recorded XRD patterns)

**Catalyst type**	**M/PEG**	**M/PEG/FeO**	**M/PEG/FeC**
d_{XRD} (nm)	22.9	21.8	19.0

Evaluation of catalytic activity of the studied catalysts

Identification of BPA degradation products during its oxidation. In our study, some intermediates (1,4-hydroquinone and phenol) were detected by HPLC-DAD in very low

concentration during Bisphenol A (BPA) conversion, when using a BPA solution with 0.5 μmol L^{-1} initial concentration.

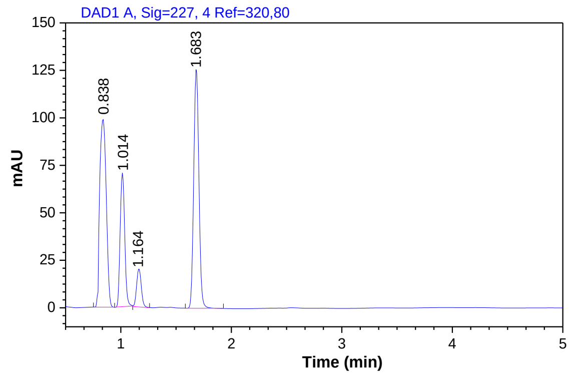

a)

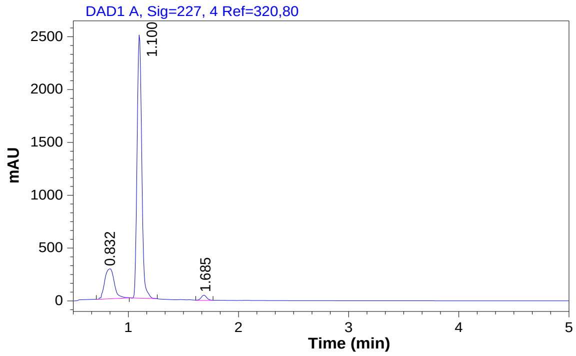

b)

Figure S3. (a) HPLC-DAD Chromatogram of a mix of standard solutions (50 μmol L^{-1} concentration) of BPA and some of its degradation products (t_R = 0.838 min – 1,4-hydroquinone; t_R = 1.014 min – 1,4-benzoquinone; t_R = 1.164 min – phenol; t_R = 1.683 min – BPA); **(b)** Chromatogram of BPA degradation kinetics with the formation of intermediate products (t_R = 0.832 min – 1,4-hydroquinone, t_R = 1.100 min – phenol, t_R = 1.685 min – BPA). Initial conditions: 50 μmol L^{-1} of BPA, 100 g L^{-1} of catalyst, pH 6.6, t = 25 °C, reaction time 120 min. HPLC-DAD (Agilent 1200 Infinity Series) method: Separations were carried out with a *Poroshell EC-C18* column (dimensions 4.6×50 mm, 2.7 μm; Agilent Technologies., USA). The injection volume was 20 μL. The mobile phase was a mixture of Formic Acid 25 mM (A) and HPLC-grade AcN (B) (50:50 v/v) at a flow rate of 0.7 mL min^{-1} under isocratic conditions.

In order to better identify the possible occurrence of intermediates during BPA oxidation, HPLC measurements were carried out on pollutant aqueous solutions with an increased concentration by

two orders of magnitude. Standard solutions (50 μmol L^{-1}) of some of the BPA established intermediate products (1,4-hydroquinone, 1,4-benzoquinone and phenol) have been freshly prepared. Then, a mixture of 50 μmol L^{-1} containing BPA and these three intermediates was further submitted to HPLC analysis in order to identify the compounds' corresponding retention times (t_R) (Fig. S3a): t_R = 0.838 min – 1,4-hydroquinone; t_R = 1.014 min – 1,4-benzoquinone; t_R = 1.164 min – phenol; t_R = 1.683 min – Bisphenol A. These peaks (Fig. S3a) were then compared with the ones obtained following the kinetic tests of BPA degradation (Fig. S3b) performed in the following reaction conditions: 50 μmol L^{-1} of BPA, 100 g L^{-1} of M/PEG/FeO catalyst, pH 6.6, t = 25 °C, reaction time 120 min, which represent the proportionally increased optimized operational parameters previously established in our present study (Fig. 3c). As it can be seen from the chromatogram in Fig. S3, in these conditions, during the BPA degradation in the absence of H_2O_2 and UV-A, such by-products have been identified: i.e. 1,4-hydroquinone at t_R = 0.832 min and phenol at t_R = 1.100 min.

Cytotoxicity assessment of BPA intermediates

The cytotoxic effects of the intermediates were assessed using a *MTT* cell viability assay.

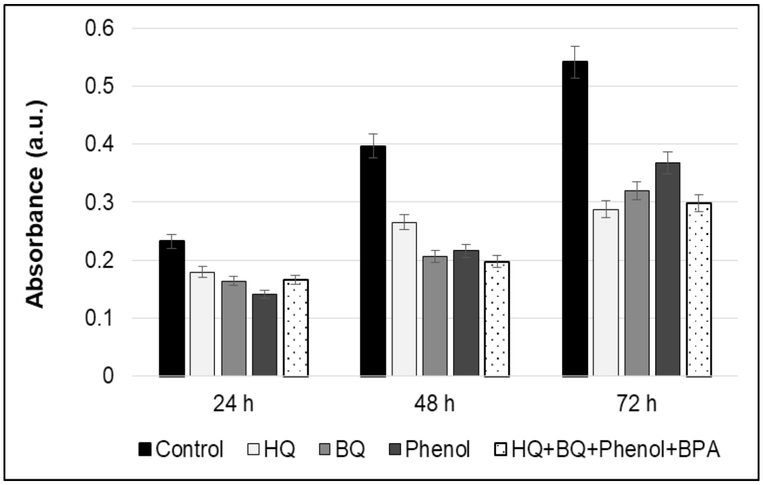

Figure S4. Cytotoxic assessment in the presence of standard 0.5 μmol L^{-1} pollutant solution, dilution of ¼: black – control; light grey – 1,4-hydroquinone (HQ); medium grey – 1,4-benzoquinone (BQ); dark grey – phenol; dotted column – mix of standard intermediates and BPA.

The effect of BPA by-products (standard solutions of either individual intermediate product: phenol, 1,4-hydroquinone, 1,4-benzoquinone or their mix together with BPA) was investigated

(Fig. S4) in order to compare the cytotoxicity level induced by the resulted intermediates during BPA conversion in the sample treatment process.

Determination of total iron in solution during BPA oxidation

The results of total iron levels measurements in solution during BPA degradation process during 120 minutes are shown in Fig. S5.

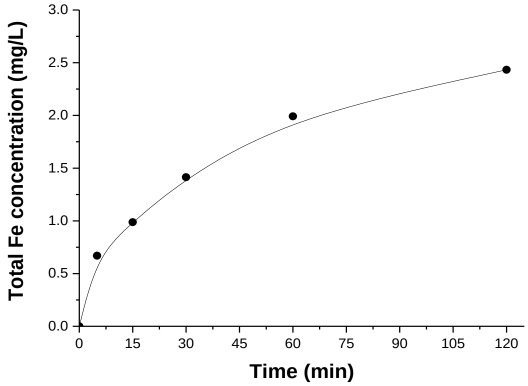

Figure S5. The total iron concentration levels in aqueous sample during BPA degradation process in time. Initial conditions: 0.5 μmol L^{-1} of BPA, 1.0 g L^{-1} of M/PEG/FeO, pH 6.6, t = 25 °C.

References

1 Nadejde, C. *et al.* Hybrid iron-based core-shell magnetic catalysts for fast degradation of bisphenol A in aqueous systems. *Chemical Engineering Journal* **302**, 587-594, doi:10.1016/j.cej.2016.05.090 (2016).

2 Mukhopadhyay, A., Joshi, N., Chattopadhyay, K. & De, G. A Facile Synthesis of PEG-Coated Magnetite (Fe3O4) Nanoparticles and Their Prevention of the Reduction of Cytochrome C. *Acs Applied Materials & Interfaces* **4**, 142-149, doi:10.1021/am201166m (2012).

3 Junejo, Y., Baykal, A. & Sozeri, H. Simple hydrothermal synthesis of Fe3O4-PEG nanocomposite. *Central European Journal of Chemistry* **11**, 1527-1532, doi:10.2478/s11532-013-0281-9 (2013).

4 Larsen, E. K. U. *et al.* Accumulation of magnetic iron oxide nanoparticles coated with variably sized polyethylene glycol in murine tumors. *Nanoscale* **4**, 2352-2361, doi:10.1039/c2nr11554a (2012).

5 Park, J. Y., Daksha, P., Lee, G. H., Woo, S. & Chang, Y. M. Highly water-dispersible PEG surface modified ultra small superparamagnetic iron oxide nanoparticles useful for target-specific biomedical applications. *Nanotechnology* **19**, doi:10.1088/0957-4484/19/36/365603 (2008).
